# Adequacy of Usual Intake of Japanese Children Aged 3–5 Years: A Nationwide Study

**DOI:** 10.3390/nu10091150

**Published:** 2018-08-23

**Authors:** Kentaro Murakami, Hitomi Okubo, M. Barbara E. Livingstone, Aya Fujiwara, Keiko Asakura, Ken Uechi, Minami Sugimoto, Han-Chieh Wang, Shizuko Masayasu, Satoshi Sasaki

**Affiliations:** 1 Department of Social and Preventive Epidemiology, School of Public Health, University of Tokyo, Tokyo 113-0033, Japan; stssasak@m.u-tokyo.ac.jp; 2 Department of Health Promotion, National Institute of Public Health, Saitama 351-0197, Japan; okubo.h.aa@niph.go.jp; 3 Nutrition Innovation Centre for Food and Health (NICHE), School of Biomedical Sciences, Ulster University, Coleraine BT52 1SA, UK; mbe.livingstone@ulster.ac.uk; 4 Department of Social and Preventive Epidemiology, Graduate School of Medicine, University of Tokyo, Tokyo 113-0033, Japan; fujiwaraay-tky@umin.ac.jp (A.F.); msugimoto@m.u-tokyo.ac.jp (M.S.); hanchieh-w@m.u-tokyo.ac.jp (H.-C.W.); 5 School of Medicine, Toho University, Tokyo 143-8540, Japan; keiko.asakura@med.toho-u.ac.jp; 6 Faculty of Health Science, Toho University, Chiba 274-8510, Japan; ken.uechi@hs.toho-u.ac.jp; 7 Ikurien-Naka, Ibaraki 311-0105, Japan; masayasu@ikurien.com

**Keywords:** children, nutrient adequacy, recommended intakes, usual nutrient intake

## Abstract

We assessed the adequacy of nutrient intakes of Japanese children aged 3–5 years. Dietary information was collected using a 3-nonconsective-day weighed dietary record among 143 boys and 143 girls. Estimates of usual nutrient intakes from foods and beverages were obtained using the best-power method. The proportions of children with intakes below and above the Japanese Dietary Reference Intakes (DRIs) were estimated. The estimated usual intakes of boys and girls were adequate for most nutrients, given the low proportion (≤2%) of children consuming diets that fell below the Estimated Average Requirement (EAR) or the mean usual intakes higher than the Adequate Intake. However, a high percentage of boys and girls had usual intakes below the EAR for calcium (64% and 54%, respectively), vitamin A (20% and 11%, respectively), thiamin (15% and 41%, respectively), and iron (27% and 10%, respectively). Additionally, 62% of boys and 66% of girls had diets that exceeded the recommended range for total fat (20%–30% energy). Further, 92% for boys and 85% for girls consumed diets that exceeded the recommended limit for sodium (4.0 and 4.5 g NaCl equivalent/day, respectively). In conclusion, the estimated usual intakes were adequate for most nutrients in this sample of 3–5-year-old Japanese children.

## 1. Introduction

Dietary habits and eating behaviors in childhood potentially have short- and long-term effects on health, including the development of many chronic diseases such as obesity [1] and cardiovascular disease [2]. Additionally, evidence suggests that dietary patterns established during childhood persist into adolescence and adulthood to some extent at least [3,4,5]. Thus, monitoring children’s dietary intake is a high public-health priority to ensure that effective health-education programs and campaigns can be implemented. For this purpose, in Western countries, Dietary Reference Intakes (DRIs) have been used to assess whether children’s diets provide enough nutrients to meet requirements without being excessive [6,7,8,9,10].

In Japan, there is a lack of information on the adequacy of nutrient intake of preschool children. The National Health and Nutrition Survey has assessed dietary intake continuously since 1945 on representative samples of Japanese aged ≥1 year [11]. However, only 1-day dietary data (at the household level) have been collected, based on which usual intake distribution cannot be estimated and thus the adequacy of nutrient intake cannot be estimated. In contrast to dietary patterns in Western countries, recent Japanese diets typically include high intakes of refined grains (mainly white rice), seaweeds, vegetables, fish, soybean products, and green tea, as well as low intakes of whole grains, processed meat, nuts, and soft drinks, at least for adults [12,13]. Nevertheless, dietary intake patterns vary depending on age [13]. Among Japanese preschool children, evaluation of contemporary nutrient intake will aid identification of potential issues of public-health concern and inform nutrition policies aimed at promoting the establishment of healthy eating patterns and dietary guidelines.

The purpose of this study was to provide estimates of usual intakes of macronutrients and micronutrients from food and beverage sources for preschool children in Japan based on dietary data obtained using a 3-day weighed dietary record, and to evaluate the adequacy of nutrient intakes in comparison with the Japanese DRIs [14].

## 2. Materials and Methods

### 2.1. Study Population

This analysis was based on data obtained from the DONGuRI (Dietary Observation and Nutrient intake for Good health Research in Japanese young children) study, a nationwide cross-sectional study. The main purpose was to describe dietary and lifestyle characteristics and investigate the associations between these characteristics and health status in two different populations, namely infants (aged 1.5–2 years) and preschool children (aged 3–6 years). For this, different assessments were conducted between these two populations. The target population comprised apparently healthy boys and girls aged 19–83 months living in private households in Japan. Initially, 24 (of 47) prefectures were selected on the basis of geographical diversity and feasibility of the survey, particularly for the recruitment of collaborators (research dietitians). A total of 323 research dietitians, many of whom worked in nursery facilities, agreed to support the study (data collection). They then conducted the recruitment of participants through nursery facilities. Based on feasibility and human and financial resources (assuming 2–3 children per research dietitian), we decided to include 16 boys and 16 girls in each prefecture (768 in total): 4 aged 19–23 months, 4 aged 24–35 months, 2 aged 36–47 months (3 years), 2 aged 48–59 months (4 years), 2 aged 60–71 months (5 years), and 2 aged 72–83 months (6 years). Note that we did not conduct specific sample-size calculation in this study. We excluded from recruitment children who were consuming special diets (e.g., vegetarian), those planning to move to a different nursery facility within a few months, and those whose guardians were dietitians or medical doctors. Participation of only one child per household was permitted. Consequently, a total of 753 children from 315 nursery facilities in 24 prefectures agreed to participate and completed all the measurements. The survey was conducted from October to December 2015.

In the Japanese DRIs [14], reference values are available for each sex and age group (e.g., boys and girls aged 1–2, 3–5, and 6–7 years) separately. In this study, only 1-day dietary data were collected for children aged 19–23 and 24–35 months, based on which usual intake could not be estimated. The number of boys and girls aged 6 years was relatively small (*n* = 43 and 47, respectively) and was considered insufficient for the purpose of usual intake estimation and comparison with reference values. We therefore focused on those aged 3–5 years. The present analysis included boys and girls aged 3–5 years with a complete dataset (3-day dietary record and anthropometric measurement; *n* = 143 for each sex).

This study was conducted according to the guidelines laid down in the Declaration of Helsinki and all procedures involving human subjects were approved by the Ethics Committee of the University of Tokyo Faculty of Medicine. Written informed consent was obtained from each participant’s parent or guardian.

### 2.2. Dietary Assessment

Dietary data were collected using a 3-nonconsecutive-day weighed dietary record, which included two weekdays (with lunch at nursery facilities on both days) and one weekend day (without lunch at nursery facilities). Research dietitians, who were responsible for the assessment of dietary intake at nursery facilities, were provided with recording sheets as well as the study manual and asked to weigh and record all foods and beverages the children consumed at nursery facilities on the recording days, in addition to any leftovers. They were encouraged to provide as much information as possible, including the names of dishes, names of foods, and ingredients in dishes, and whether the foods were prepared at nursery facilities or were ready-made.

In a similar manner, dietary recording for foods and beverages consumed out of nursery facilities was conducted by a guardian of each child (in almost all cases the mother). The research dietitian gave each parent or guardian an oral explanation of the diet-recording method, a digital kitchen scale (KD-812WH; Tanita, Tokyo, Japan), a measuring spoon, a measuring cup, a handbook for the dietary record, and recording sheets. The guardians were asked to weigh and record all food and beverage items consumed by the children. On occasions when weighing was problematic (e.g., dining out), they were instructed to document as much information as possible, including the brand name of the food and the consumed portion size (based on typical household measures), as well as the details of leftovers. On the weekday following each recording day, the recording form as well as packaging for any processed foods consumed were handed to the research dietitian, who reviewed the forms and, whenever necessary, sought additional information or modification of the record.

As requested in the protocol for dietary data processing specifically developed for this study, research dietitians converted the estimates of portion sizes recorded using household measures into weights, and coded all individual food items on the basis of the Standard Tables of Food Composition in Japan [15]. Food items not in the tables were assigned the nutrient values of similar food items. The data were entered into an Excel spreadsheet specially designed for this study. All the spreadsheet files were compiled in the central office, where two dietitians checked all the recorded food items and weights and modified them if needed. Estimates of daily intakes of energy and nutrients were calculated using the Standard Tables of Food Composition in Japan [15]. We did not consider dietary supplements during the nutrient intake calculation not only because the use of dietary supplements during the previous month was rare in this population (3.5%) but also because of the lack of a dietary-supplement database in Japan. Thus, it was our intention to consider only nutrient intake from foods and beverages, in accordance with the purpose of the Japanese DRIs [14].

### 2.3. Handling of Dietary Misreporting

Misreporting of energy intake (EI) was evaluated on the basis of the ratio of EI to basal metabolic rate (BMR) (the Goldberg cut-off) [16] and the ratio of EI to estimated energy requirement (EER), namely the procedure proposed by Huang and colleagues [17]. Body height (to the nearest 0.1 cm) and weight (to the nearest to 0.1 kg) were measured in light indoor clothing and no shoes, either as part of this study or during a routine health check-up (within one month before the study period). BMR was estimated using the Henry sex- and age-specific equations based on body height and weight [18]. Assuming the physical activity level (PAL) of a sedentary lifestyle (i.e., 1.55) [16] (because of the lack of an objective measure of physical activity in the present study), under-reporters, plausible reporters, and over-reporters were defined as having an EI:BMR of <1.00, 1.00–2.40, and >2.40, respectively [16]. EER was calculated using sex- and age-specific equations published in the US/Canada DRIs, based on sex, age, body height and weight, and physical activity [19]. During this calculation, we assumed a ‘low-active’ level of physical activity (i.e., PAL ≥ 1.4 to <1.6) [19], again owing to the lack of an objective measure of physical activity. Taking into account the number of recording days (3 days) and the coefficient of variation in intakes and other components of energy balance (i.e., within-subject variation in EI, 23%; error in the EER equations, 6.5%; and day-to-day variation in total energy expenditure, 8.2%) [16,17], under-reporters, plausible reporters, and over-reporters were defined as having an EI:EER of <0.71, 0.71–1.40, and >1.40, respectively. Misreporters were thus identified in this study but were not excluded from the analysis, on the basis that exclusion would have introduced bias [20,21].

### 2.4. Estimation of Usual Intakes

Usual intakes of nutrients were computed according to the best-power method proposed by Nusser and colleagues [22,23]. Briefly, this method uses ANOVA to estimate the usual intake of ubiquitously consumed nutrients by correcting for the within-person variation in nutrient intake across days, after normalizing the distribution of nutrient intake by Box–Cox transformation. The best-power method was implemented using the HabitDist software [24], which was designed for this purpose. This program estimated the mean (SD) and distribution (10th, 25th, 50th, 75th, and 90th percentiles) of usual daily-nutrient intakes (as well as the proportion above or below the defined cut-off values).

### 2.5. Assessment of Nutrient Intake Adequacy

To assess nutrient-intake adequacy, estimated usual nutrient intakes were compared with age- and sex-specific reference values in the latest Japanese DRIs (i.e., DRIs for Japanese, 2015) [14] to compute the percentage of children meeting the DRIs. Of the total of 33 nutrients presented in the DRI, 5 nutrients (i.e., biotin, iodine, selenium, chromium, and molybdenum) were excluded from the present analysis because of the lack of sufficient food-composition tables for these in Japan [15]. DRIs include several reference values. The Estimated Average Requirement (EAR) is “the average daily-nutrient-intake level estimated to meet the requirements of half the healthy individuals in a particular life stage and gender group” [25]. Intakes lower than the EAR indicate the estimated prevalence of inadequate intakes within a group. The Adequate Intake (AI) is “a recommended average daily-nutrient-intake level based on observed or experimentally determined approximations or estimates of nutrient intake by a group (or groups) of apparently healthy people that are assumed to be adequate” [25]. AI is used when EAR cannot be determined [25]. For nutrients with an AI rather than an EAR, the group mean intake is compared with the AI, and a group mean intake at or above the AI implies that the prevalence of inadequacy is low [26]. If a group’s mean intake is below the AI, intakes may need to increase, but it is not possible to precisely quantify the prevalence of inadequacy [26]. We calculated the percentage of children consuming less than the EAR (for protein, vitamin A, thiamin, riboflavin, niacin, vitamin B-6, vitamin B-12, folate, vitamin C, calcium, magnesium, iron, zinc, and copper) and greater than or equal to the AI (for n-6 PUFA, n-3 PUFA, vitamins D, E, and K, pantothenic acid, potassium, phosphorus, and manganese). The Japanese DRIs have the Tolerable Upper Intake Level (UL) of several nutrients for boys and girls aged 3–5 years (i.e., vitamins A, D, E, and B-6, niacin, folate, and iron). Note that none of the children in this study had intakes above the UL of any nutrient.

In the Japanese DRIs [14], a Tentative Dietary Goal for Preventing Lifestyle-related Diseases (DG) is given for macronutrient balance (% of energy from protein, total fat, SFA, and carbohydrate), dietary fiber, sodium, and potassium. The DG is “the average daily-nutrient-intake level (or ranges) that Japanese should currently aim to consume primarily to prevent chronic diseases” [14]. It should be noted that all the Japanese DRIs for boys and girls aged 3–5 years are based on moderate level of physical activity. For boys and girls aged 3–5 years, a DG is not available for SFA, dietary fiber, and potassium. We tentatively applied the DG for SFA in adults to children, in accordance with a previous study [27]. For dietary fiber and potassium, we determined tentative DGs for boys and girls aged 3–5 years according to a procedure that was applied to determine these DGs for older children [14], mainly based on the median intake in the National Health and Nutrition Survey 2010 and 2011 [13]. We calculated the percentage of children consuming nutrient intake outside (below or above) the range of the DG.

## 3. Results

This analysis included 143 boys and 143 girls aged 3–5 years (Table 1). The 3-day mean EI was on average 5761 kJ/day (1377 kcal/day) and 5414 kJ/day (1294 kcal/day) for boys and girls, respectively. The mean EI:BMR and EI:EER were quite close to the PAL for sedentary lifestyle (1.55) and the expected ratio of 1.00, respectively, in both sexes. The use of the Goldberg cut-off (based on EI:BMR) identified only one under-reporter (girl), while based on the EI:EER, there were four under-reporters (one boy and three girls) and three over-reporters (two boys and one girl). These findings suggest satisfactory reporting accuracy of dietary intake.

The usual intake distributions of energy and macronutrients for boys and girls are shown in Table 2 and Table 3, respectively. For protein, no boys or girls had usual intakes below the EAR. In boys, mean usual intake of n-6 PUFA was below the AI, while that of n-3 PUFA was above the AI; in girls, mean usual intakes were above the AI for both nutrients. An estimated 59% of boys and 39% of girls had diets that fell below the (tentative) DG for dietary fiber. For macronutrient balance, 62% of boys and 66% of girls had diets that exceeded the DG for total fat, with a low proportion of children consuming diets that fell outside the range of DG for protein and carbohydrate (≤8%). For SFA, almost all children (≥98%) exceeded the (tentative) DG.

We also compared the usual intakes of vitamins and minerals to the corresponding DRIs for boys and girls (Table 4 and Table 5, respectively). For riboflavin, niacin, vitamins B-6, B-12, and C, folate, magnesium, zinc, and copper, ≤2% of boys and girls consumed diets that fell below the EAR. Conversely, a high percentage of boys and girls had usual intakes below the EAR for calcium (64% and 54%, respectively), vitamin A (20% and 11%, respectively), thiamin (15% and 41%, respectively), and iron (27% and 10%, respectively). For nutrients with AI, mean usual intakes were higher than the AI except for phosphorus in boys. For potassium, 22% of boys and 10% of girls had diets that fell below the (tentative) DG. Further, usual intakes of sodium exceeded the DG in 92% of boys and 85% of girls.

## 4. Discussion

To our knowledge, this is the first study to estimate usual intakes of macronutrients and micronutrients from food and beverage sources for preschool children in Japan and to evaluate the adequacy of nutrient intakes. The present findings should serve as a reference on nutrient intake and adequacy in Japanese children aged 3–5 years for future research and nutrition-policy development. In comparison with the Japanese DRIs [14], the estimated usual intakes in this sample were adequate for most nutrients, including protein, riboflavin, niacin, vitamins B-6, B-12, and C, folate, magnesium, zinc, and copper (assessed based on EAR) as well as *n*-6 PUFA (girls only), *n*-3 PUFA, vitamins D, E, and K, pantothenic acid, potassium, phosphorus (girls only), and manganese (assessed based on AI). This is generally consistent with previous findings in Japanese children attending elementary school (aged 8–9 and 10–11 years) and junior high school (aged 13–14 years) [28]. The results were generally similar for both boys and girls, although there were slight differences in several nutrients (for example, the percentage of children whose usual intakes below the EAR for iron was somewhat higher in boys (27%) than in girls (10%), which may be mainly due to the higher EAR value in boys than in girls). Based on the use of tentative reference values, inadequate intake of dietary fiber and potassium and an excessive intake of SFA were also evident. The previous study in older children also found inadequate intakes of these nutrients in at least some age and sex groups [28]. Thus, these findings may reflect dietary characteristics (major dietary patterns) pervasive across a wide range of age groups in Japan. Nevertheless, given that the Japanese DRI values for children are generally derived by extrapolating the values for adults, it is possible that the Japanese DRIs for children are inappropriate (too high), for some nutrients at least. Accordingly, to better inform the Japanese DRIs for children, we should await the accumulation of detailed information on nutrient intake.

Generally consistent with these findings in Japan, usual intakes of most nutrients are also adequate in young children in developed Western countries [6,7,8,9,10]. However, intake of nutrients of concern seems to vary across countries, except for inadequate intakes of vitamin D, which are ubiquitous [6,7,8,9,10]. For example, in the United States, concern has been expressed over a high intake of sodium and low intakes of potassium, dietary fiber, and SFA [7]. Usual intakes of vitamin E, folate, and calcium were insufficient in Spanish children [8], while total-fat and SFA intakes exceeded the reference ranges [9]. The diets of Irish preschoolers were high in sodium and low in EPA, DHA, iron, calcium, and zinc, in comparison with nutrient recommendations [10]. These differences may be mainly due to differences in dietary patterns as well as in the reference values used. For example, adequate intake of vitamin D observed here, which contrasts with the findings in Western countries, is mainly due to low reference values in the Japanese DRIs rather than to high intakes. In any case, these findings clearly indicate that improving the nutrient intake of children requires different strategies in different countries.

Several limitations of this study are acknowledged. First, our participants were not a representative sample of the general Japanese population but rather volunteers, and thus the guardians of our participants were possibly health-conscious. Mean height and weight were, however, reasonably comparable with those observed in a national representative sample [29]. Furthermore, the present sample exclusively comprised children attending nursery facilities, despite the fact that only around half of children aged 3–6 years receive nursery service in Japan [30]. Given that the foods and dishes provided in nursery facilities (mainly lunch) are planned and controlled on the basis of a guideline that is based on the Japanese DRIs [31] (while there is no relevant nutrition policy for preschool children not attending nursery facilities), it may be likely that the nutrient intake of children attending nursery facilities is on average better than that of other children. If so, this study may overestimate nutritional adequacy (and underestimate nutritional inadequacy), and thus the generalizability of the present findings may be limited. Research among a more representative sample is needed.

Second, all self-reported dietary assessment methods are subject to both random and systematic measurement errors [20]. Dietary records are generally susceptible to measurement errors due to erroneous recording and potential changes in eating behavior [32], although it is currently unknown if or to what extent such phenomena are relevant in small children. To minimize potential systematic errors, research dietitians recorded food intake in nursery facilities and checked dietary information recorded by guardians (out of nursery facilities), in accordance with the study protocol. In fact, given that only a few energy misreporters were identified, and that mean EI:BMR and EI:EER were relatively close to the expected ratios, we are confident that diet reporting bias has been minimized. However, because the survey was conducted within a three-month period (from October to December), any seasonal variation in nutrient intake was not considered. This might have produced some bias in assessing average intake over the year, given that several previous studies have observed seasonal differences in intakes of at least some nutrients in Japanese adults [33,34,35]. Further studies on this question in children are needed. Finally, as a general limitation, the food-composition tables may not necessarily reflect the true nutrient composition of the foods, which vary due to differences in, for example, season and variety. It should be noted, however, that we used the latest Standard Tables of Food Composition in Japan [15], the most comprehensive and relevant tables available.

## 5. Conclusions

When compared with the current Japanese DRIs [14], the estimated usual intakes in this sample of Japanese children aged 3–5 years were adequate for most nutrients, namely protein, riboflavin, niacin, vitamins B-6, B-12, and C, folate, magnesium, zinc, and copper (assessed based on EAR), as well as n-6 PUFA (girls only), n-3 PUFA, vitamins D, E, and K, pantothenic acid, potassium, phosphorus (girls only), and manganese (assessed based on AI). However, we also found inadequate intakes of calcium, vitamin A, thiamin, and iron, and excess intakes of total fat (% energy) and sodium. These findings should prove useful in the development of nutrition policies as well as dietary guidelines. The public health nutrition issues identified here warrant further investigation, including dietary sources associated with inadequate and excessive intakes.

## Figures and Tables

**Table 1:** Basic characteristics of participants.

	Boys (*n =* 143)		Girls (*n =* 143)	
	Mean	SD	Mean	SD
Age, *n* (%)				
3 years	47 (32.9)		47 (32.9)	
4 years	48 (33.6)		48 (33.6)	
5 years	48 (33.6)		48 (33.6)	
Body height (cm)	103.5	7.3	102.6	6.9
Body weight (kg)	16.7	2.7	16.6	2.5
BMI (kg/m^2^)	15.5	1.2	15.7	1.1
EI (kJ/day) ^a^	5761	908	5414	920
BMR (kJ/day)	3694	259	3464	222
EER (kJ/day)	5945	510	5590	343
EI:BMR ^a^	1.56	0.2	1.56	0.23
EI:EER ^a^	0.97	0.13	0.97	0.14

BMI, body mass index; BMR, basal metabolic rate; EER, estimated energy requirement; EI, energy intake. ^a^ EI calculated as a three-day mean per participant was used.

**Table 2:** Usual intakes of energy and macronutrients from foods and beverages for Japanese boys aged 3–5 years (*n* = 143) ^a^.

	Mean	SD	Percentile					DRI Value ^b^			DRI Compliance (%)			
			10th	25th	50th	75th	90th	EAR	AI	DG	<EAR	≥AI	<DG	>DG
Energy (kJ/day)	5761	753	4833	5222	5699	6134	6816	---	---	---	---	---	---	---
Macronutrients														
Protein (g/day)	48.7	7.3	39.7	43.3	48.4	54.0	58.0	20	---	---	0	---	---	---
Protein (g/day per kg body weight)	2.95	0.71	2.06	2.42	2.90	3.43	3.85	---	---	---	---	---	---	---
Fat (g/day)	45.3	6.1	37.8	40.6	44.7	49.8	53.8	---	---	---	---	---	---	---
SFA (g/day)	15.2	2.5	12.5	13.2	14.8	17.2	18.3	---	---	---	---	---	---	---
*n*-6 PUFA (g/day)	6.7	1.0	5.3	6.0	6.7	7.3	8.2	---	7	---	---	32.2	---	---
*n*-3 PUFA (g/day)	1.4	0.3	1.0	1.2	1.4	1.6	1.9	---	1.3	---	---	60.1	---	---
Carbohydrate (g/day)	190.3	27.5	159.4	170.3	188.6	202.9	232.1	---	---	---	---	---	---	---
Dietary fiber (g/day)	8.8	1.7	6.6	7.7	8.7	9.2	11.3	---	---	≥9 ^c^	---	---	58.7	---
Macronutrient balance														
Protein (% energy)	14.2	0.7	13.2	13.7	14.2	14.7	15.1	---	---	13–20	---	---	6.3	0
Fat (% energy)	29.3	2.3	26.4	27.9	29.2	30.8	32.0	---	---	20–30	---	---	0	61.5
SFA (% energy)	9.9	1.4	8.1	8.9	9.8	10.8	11.8	---	---	≤7 ^d^	---	---	---	98.6
Carbohydrate (% energy)	55.5	2.8	51.9	53.7	55.5	57.3	59.4	---	---	50-65	---	---	2.1	0

AI, Adequate Intake; DG, Tentative Dietary Goal for Preventing Lifestyle-related Diseases; DRI, Dietary Reference Intake; EAR, Estimated Average Requirement. ^a^ Usual intake was estimated using the best-power method based on three-day weighed dietary-record data. ^b^ DRI values are from DRIs for Japanese, 2015. ^c^ The DG for this age group was not determined in the Japanese DRIs. For this analysis, we determined the tentative DG according to the procedure suggested in the Japanese DRIs (mainly based on the median value of dietary fiber intake in the National Health and Nutrition Survey 2010 and 2011) [13]. ^d^ The DG for this age group was not determined in the Japanese DRIs. For this analysis, we tentatively used the DG for adults aged ≥18 years.

**Table 3:** Usual intakes of energy and macronutrients from foods and beverages for Japanese girls aged 3–5 years (*n* = 143) ^a^.

	Mean	SD	Percentile					DRI Value ^b^			DRI Compliance (%)			
			10th	25th	50th	75th	90th	EAR	AI	DG	<EAR	≥AI	<DG	>DG
Energy (kJ/day)	5414	749	4469	4916	5435	5958	6222	---	---	---	---	---	---	---
Macronutrients														
Protein (g/day)	45.8	5.7	38.7	42.0	45.8	48.8	53.0	20	---	---	0	---	---	---
Protein (g/day per kg body weight)	2.79	0.67	1.99	2.36	2.74	3.22	3.61	---	---	---	---	---	---	---
Fat (g/day)	42.6	7.0	34.1	38.0	41.7	47.8	51.9	---	---	---	---	---	---	---
SFA (g/day)	14.1	2.5	10.9	12.3	13.7	15.7	17.5	---	---	---	---	---	---	---
*n*-6 PUFA (g/day)	6.5	1.2	5.1	5.7	6.3	7.3	8.2	---	6	---	---	64.3	---	---
*n*-3 PUFA (g/day)	1.4	0.3	1.0	1.2	1.4	1.6	1.8	---	1.1	---	---	87.4	---	---
Carbohydrate (g/day)	178.7	27.0	144.6	158.7	179.7	194.9	207.8	---	---	---	---	---	---	---
Dietary fiber (g/day)	8.7	1.7	6.7	7.4	8.5	9.7	11.2	---	---	≥8 ^c^	---	---	38.5	---
Macronutrient balance														
Protein (% energy)	14.3	1.0	13.0	13.5	14.2	14.9	15.5	---	---	13–20	---	---	8.4	0
Fat (% energy)	29.2	2.2	26.3	27.8	29.3	30.6	32.2	---	---	20–30	---	---	0	65.7
SFA (% energy)	9.6	1.2	8.0	8.8	9.6	10.5	11.3	---	---	≤7 ^d^	---	---		100
Carbohydrate (% energy)	55.5	2.6	51.9	53.6	55.5	57.2	58.8	---	---	50–65	---	---	1.4	0

AI, Adequate Intake; DG, Tentative Dietary Goal for Preventing Lifestyle-related Diseases; DRI, Dietary Reference Intake; EAR, Estimated Average Requirement. ^a^ Usual intake was estimated using the best-power method based on three-day weighed dietary-record data. ^b^ DRI values are from DRIs for Japanese, 2015. ^c^ The DG for this age group was not determined in the Japanese DRIs. For this analysis, we determined the tentative DG according to the procedure suggested in the Japanese DRIs (mainly based on the median value of dietary fiber intake in the National Health and Nutrition Survey 2010 and 2011) [13]. ^d^ The DG for this age group was not determined in the Japanese DRIs. For this analysis, we tentatively used the DG for adults aged ≥18 years.

**Table 4:** Usual intakes of vitamins and minerals from foods and beverages for Japanese boys aged 3–5 years (*n* = 143) ^a^.

	Mean	SD	Percentile					DRI Value ^b^			DRI Compliance (%)			
			10th	25th	50th	75th	90th	EAR	AI	DG	<EAR	≥AI	<DG	>DG
Vitamins														
Vitamin A (μg RAE/day) ^c^	392	51	330	357	390	423	457	350	---	---	20.3	---	---	---
Vitamin D (μg/day)	4.2	1.5	2.5	3.1	4.0	5.1	6.0	---	2.5	---	---	90.2	---	---
Vitamin E (mg/day)	5.0	0.9	3.8	4.2	4.9	5.5	6.2	---	4.5	---	---	64.3	---	---
Vitamin K (μg/day)	131.8	27.8	98.2	113.4	125.3	147.9	167.9	---	70	---	---	100	---	---
Thiamin (mg/day)	0.68	0.08	0.58	0.63	0.69	0.73	0.78	0.6	---	---	15.4	---	---	---
Riboflavin (mg/day)	0.95	0.15	0.77	0.83	0.94	1.03	1.18	0.7	---	---	2.1	---	---	---
Niacin (mg NE/day) ^d^	18.0	3.1	14.0	15.7	17.7	20.2	22.3	6	---	---	0	---	---	---
Vitamin B-6 (mg/day)	0.85	0.14	0.68	0.76	0.84	0.95	1.06	0.5	---	---	0	---	---	---
Vitamin B-12 (μg/day)	3.9	1.3	2.5	3.0	3.6	4.6	5.4	0.8	---	---	0	---	---	---
Folate (μg/day)	190	37	142	162	188	216	245	80	---	---	0	---	---	---
Pantothenic acid (mg/day)	4.6	0.7	3.8	4.2	4.6	5.0	5.5	---	4	---	---	80.4	---	---
Vitamin C (mg/day)	71.0	22.8	46.1	52.7	67.8	85.0	104.2	35	---	---	1.4	---	---	---
Minerals														
Sodium (g NaCl equivalent/day) ^e^	5.7	1.2	4.1	4.9	5.5	6.4	7.3	---	---	<4.0	---	---	---	92.3
Potassium (mg/day)	1785	248	1415	1637	1782	1944	2133	---	1100	≥1600 ^f^	---	100	22.4	---
Calcium (mg/day)	483	98	373	413	473	536	634	500	---	---	64.3	---	---	---
Magnesium (mg/day)	174	25	141	156	171	188	211	80	---	---	0	---	---	---
Phosphorus (mg/day)	793	114	649	705	784	858	957	---	800	---	---	44.8	---	---
Iron (mg/day)	4.6	0.8	3.6	4.0	4.5	5.1	5.6	4.0	---	---	26.6	---	---	---
Zinc (mg/day)	6.0	0.8	5.0	5.5	6.0	6.4	7.0	3	---	---	0	---	---	---
Copper (mg/day)	0.74	0.11	0.64	0.07	0.73	0.80	0.88	0.3	---	---	0	---	---	---
Manganese (mg/day)	1.8	0.4	1.3	1.5	1.7	2.1	2.3	---	1.5	---	---	76.2	---	---

AI, Adequate Intake; DG, Tentative Dietary Goal for Preventing Lifestyle-related Diseases; DRI, Dietary Reference Intake; EAR, Estimated Average Requirement; NE, niacin equivalent; RAE, retinol activity equivalent. ^a^ Usual intake was estimated using the best-power method based on three-day weighed dietary-record data. ^b^ DRI values are from DRIs for Japanese, 2015. ^c^ 1 μg RAE = sum of retinol (μg) + β-carotene (μg) × 1/12 + α-carotene (μg) × 1/12 + β-cryptoxanthin (μg) × 1/24. ^d^ 1 mg NE = niacin (mg) + protein (mg)/6000. ^e^ 1 g NaCl equivalent = 58.5/23 × sodium (g). ^f^ The DG for this age group was not determined in the Japanese DRIs. For this analysis, we determined the tentative DG according to the procedure suggested in the Japanese DRIs (mainly based on the median value of dietary fiber intake in the National Health and Nutrition Survey 2010 and 2011) [13].

**Table 5:** Usual intakes of vitamins and minerals from foods and beverages for Japanese girls aged 3–5 years (*n* = 143) ^a^.

	Mean	SD	Percentile					DRI Value ^b^			DRI Compliance (%)			
			10th	25th	50th	75th	90th	EAR	AI	DG	<EAR	≥AI	<DG	>DG
Vitamins														
Vitamin A (μg RAE/day) ^c^	370	61	298	329	364	412	454	300	---	---	11.2	---	---	---
Vitamin D (μg/day)	4.4	1.3	2.9	3.5	4.3	5.3	6.2	---	2.5	---	---	95.8	---	---
Vitamin E (mg/day)	4.7	0.6	4.0	4.3	4.6	5.2	5.7	---	4.5	---	---	59.4	---	---
Vitamin K (μg/day)	134.8	41.8	87.8	105.4	127.7	158.8	187.6	---	70	---	---	99.3	---	---
Thiamin (mg/day)	0.63	0.09	0.51	0.56	0.62	0.7	0.76	0.6	---	---	40.6	---	---	---
Riboflavin (mg/day)	0.88	0.13	0.72	0.79	0.87	0.99	1.05	0.6	---	---	0.7	---	---	---
Niacin (mg NE/day) ^d^	16.6	2.7	13.4	14.9	16.6	18.5	19.5	6	---	---	0	---	---	---
Vitamin B-6 (mg/day)	0.79	0.13	0.64	0.69	0.78	0.88	0.98	0.5	---	---	0	---	---	---
Vitamin B-12 (μg/day)	3.9	0.8	2.9	3.3	3.7	4.3	5.00	0.8	---	---	0	---	---	---
Folate (μg/day)	183	36	142	155	180	207	230	80	---	---	0	---	---	---
Pantothenic acid (mg/day)	4.3	0.6	3.5	3.8	4.2	4.7	5.1	---	4	---	---	62.9	---	---
Vitamin C (mg/day)	67.9	16.6	46.1	55.8	67.3	77.7	92.0	35	---	---	0	---	---	---
Minerals														
Sodium (g NaCl equivalent/day) ^e^	5.5	1.0	4.3	4.8	5.6	6.3	6.8	---	---	<4.5	---	---	---	85.3
Potassium (mg/day)	1676	237	1404	1491	1631	1830	2042	---	1000	≥1400 ^f^	---	100	9.8	---
Calcium (mg/day)	453	83	351	390	443	504	582	450	---	---	53.8	---	---	---
Magnesium (mg/day)	165	23	139	149	164	178	195	80	---	---	0	---	---	---
Phosphorus (mg/day)	744	92	634	678	739	300	865	---	600	---	---	95.1	---	---
Iron (mg/day)	4.4	0.7	3.5	3.9	4.4	4.9	5.4	3.5	---	---	9.8	---	---	---
Zinc (mg/day)	5.6	0.6	4.7	5.2	5.6	6.0	6.3	3	---	---	0	---	---	---
Copper (mg/day)	0.71	0.11	0.57	0.64	0.70	0.77	0.86	0.3	---	---	0	---	---	---
Manganese (mg/day)	1.7	0.3	1.4	1.5	1.7	1.9	2.2	---	1.5	---	---	75.5	---	---

AI, Adequate Intake; DG, Tentative Dietary Goal for Preventing Lifestyle-related Diseases; DRI, Dietary Reference Intake; EAR, Estimated Average Requirement; NE, niacin equivalent; RAE, retinol activity equivalent. ^a^ Usual intake was estimated using the best-power method based on three-day weighed dietary-record data. ^b^ DRI values are from DRIs for Japanese, 2015. ^c^ 1 μg RAE = sum of retinol (μg) + β-carotene (μg) × 1/12 + α-carotene (μg) × 1/12 + β-cryptoxanthin (μg) × 1/24. ^d^ 1 mg NE = niacin (mg) + protein (mg)/6000. ^e^ 1 g NaCl equivalent = 58.5/23 × sodium (g). ^f^ The DG for this age group was not determined in the Japanese DRIs. For this analysis, we determined the tentative DG according to the procedure suggested in the Japanese DRIs (mainly based on the median value of dietary fiber intake in the National Health and Nutrition Survey 2010 and 2011) [13].
